# Physicochemical and Microbiological Quality of Dietetic Functional Mixed Cerrado Fruit Jam during Storage

**DOI:** 10.1155/2018/2878215

**Published:** 2018-08-26

**Authors:** T. M. Brandão, E. L. do Carmo, H. E. S. Elias, E. E. N. de Carvalho, S. V. Borges, G. A. S. Martins

**Affiliations:** ^1^Department of Food Science, Federal University of Lavras (UFLA), Campus University, C.P. 3037,Lavras, Minas Gerais CEP 37200-000, Brazil; ^2^Department of Food Engineering, Federal University of Tocantins (UFT), Campus University of Palmas, Palmas, Tocantins, CEP 77001-090, Brazil

## Abstract

The objective of the research was to evaluate changes of dietetic functional mixed cerrado fruit jam (marolo, sweet passion fruit, and soursop) processed in a vacuum pot and stored for 180 days in BODs at 25°C and 35°C. The parameters evaluated were pH, soluble solids (SS), titratable acidity (TA), total sugars (TS), total carotenoids (TC), total phenolics (TP), vitamin C, antioxidant activity (DPPH), and microbiological analysis. There was a significant effect of storage time on pH, SS, TA, TC, TS, and TP. Vitamin C and DPPH showed an effect for the temperature x storage time interaction. Statistical models are not adjusted for pH and SS, presenting an average of 4.15 and 61%, respectively. Carotenoids decreased up to105 days; total sugars increased up to 105 days. The TP, vitamin C, and DPPH, at the temperatures evaluated, showed a decrease up to 105 days. Yeasts and filamentous fungi were not detected.

## 1. Introduction

There is considerable demand for fresh fruit and their products. It is known that most fruits are seasonal and their useful life is very limited life, often requiring the application of heat treatments, via processing, in order to maintain and preserve quality and provide various products such as pulp, jam, jellies, and juices throughout the year, especially in the off-season. The flora of the cerrado has various fruit species with high potential for agricultural use, which are traditionally used by local people. The fruits are usually consumed raw or in the form of juices, liquors, ice cream, jams/jellies, and various sweet formulations [1, 2]. They have pleasant flavors, high levels of sugars, proteins, minerals, fatty acids [2], B vitamins, and carotenoids [3].

However, they are perishable and the production of jam by using various additives (sugar, acid, sweetener, pectin, among others) and thermal processing is intended to prevent loss of postharvest fruit and offer a differentiated product to the consumer. According Worobo and Splittstoesser [4] vacuum processing allows the use of lower temperatures, thus resulting in less heat damage to the bioactive compounds and other sensory characteristics.

The useful life of a product is characterized in the period in which it is in good microbiological and sensory condition for consumption without jeopardize taste and health. These conditions are directly dependent on the physical, chemical, and microbiological transformations during storage, which are also related to the nature of the product (kind and amount of ingredients), packaging, and storage conditions (humidity, temperature, and storage time) [5, 6].

The effect of temperature is very complex and may be due to various causes. Initially, with increasing temperature molecular activity increases as well as the formation of enzyme complexes [7]. According to Lee et al. [8], the thermal processing of jellies and jams degrades the chemical components and reduces biological activity, but promotes a desirable increase in product shelf life.

Processing and storage of jams must be conducted in order to maintain their color, nutritional value, and radical scavenging activity [9–11].

According to Rababahet al. [11] for a jam to be considered of good quality it must generally provide bright color, distinctive flavor of the original fruit, intermediate consistency, and texture (not too runny nor too hard). However, these quality properties may be affected during storage. In the literature there are studies available on the storage of fruit jellies, such as strawberry guava with marolo [12], guava [13], banana peel [14], pineapple [15], cherry [11], and strawberry [16], among others. Despite this, few references are cited with cerrado fruits related to the storage of light and diet jams.

The objective of this study was to evaluate the physicochemical and microbiological alterations that occurred in the dietetic functional mixed cerrado fruit jam (marolo, sweet passion fruit and soursop), processed in a vacuum pot and packaged in polypropylene jars and stored in a temperature controlled chamber (BOD) at 25 and 35°C for 180 days.

## 2. Materials and Methods

### 2.1. Raw Material

The marolo (*Annona crassiflora* Mart.) and sweet passion fruit (*Passiflora alata*, Dryand) were purchased at CEASA-Contagem MG. The soursop pulp (*Annona muricata*, Linnaeus) was acquired from a commercial enterprise in the city of Ubá-MG.

### 2.2. Pulp Preparation

The marolo and sweet passion fruit were washed in running water and immersed in 150 mg L^−1^ sodium hypochlorite solution for 15 minutes. After sanitizing, the fruits were processed at the Pilot Plant Laboratory-DCA Federal University of Lavras/UFLA. The marolo pulp was extracted manually with the aid of a knife to remove the seed from the pulp. The sweet passion fruit pulp was pulped manually, with the seeds removed with a spoon. Later, pulps (marolo and sweet passion fruit) were pulsed and mixed in a blender and were then packed in polyethylene bags and stored in a freezer at -18°C for use in the preparation of the mixed jam and for further analysis.

### 2.3. Mixed Jam Processing

The preparation of mixed jam followed the formulation methodology developed according to previous results described by Souza et al. [17]. The mixed jam processing occurred in the Plant Product Pilot Plant-ITAL, Campinas, SP, and was conducted in triplicate in a pot with vacuum pressure jacket (Maincal, Rosario, Argentina) in which 60% fruit pulp mixture (20% of each pulp) and 40% other ingredients such as sweeteners, bodying agents, preservatives, and gelling agents were added as described in Table 1.

To process the mixed jam, a mixture of fruit pulp was made [marolo/sweet passion fruit/soursop (1:1:1)] and polydextrose was added. When the soluble solids reached 20°Bx, the low methoxil pectin (LMP) pectin and gum (carrageenan and locust) previously dissolved in water at 80°C were added to the first ingredients. For each 4 grams of pectin 50mL of hot water was used, when the soluble solids reached 25°Brix the fructooligosaccharides (FOS) were dissolved in water and added in a 1:1 ratio. At the end of the cooking process citric acid, potassium sorbate and sweeteners (sucralose and acesulfame-K) were added and then baking was stopped.

After processing, the jam was packaged in sterilized polypropylene jars. The filling was carried out hot (approximately 70-75°C). The containers were then closed, inverted (top down), cooled to room temperature, and then were kept under refrigeration for later analysis.

### 2.4. Experimental Design

To evaluate the effect of time x temperature of the mixed jam that was stored in a climatic chamber, BODs (ELETROLAB, Brazil), an experiment was conducted in a factorial 2 x 5, with two temperatures (25 and 35°C) and 5 storage times (0, 70, 105, 140, and 180 days) in triplicate. The analysis follows the model described below [18]:

(1) $$\begin{array}{c} Y_{ij}=\mu +\alpha_{i}+\beta_{J}+\beta_{J}^{2}+\alpha \beta_{\left(IJ\right)}^{2}+\epsilon_{ij} \end{array}$$

where

 
*μ* is constant associated with all treatments;
 
*α* is effect of the *i*^th^ storage time,
  i is 25°C and 35°C and is considered fixed;
 
*β* is effect of the *j*^th^ storage time, j = 0,  ....., 180;
 
*β*^2^ is quadratic effect of the *j*^th^ storage time;
 
*ϵ* is residue associated with the observations, being considered with ~ N (0, *σ*^2^) and other interactions.

### 2.5. Chemical, Physicochemical, and Microbiological Analysis

The analyses were carried out in triplicate in dietetic functional mixed cerrado fruit jam. pH, soluble solids, and total sugars were determined using the AOAC [19] and Instituto Adolfo Lutz [20] techniques. The total carotenoids content were determined according to the method proposed by Rodriguez-Amaya [21]. The determination was performed by the colorimetric method using 2,4-dinitrophenylhydrazine, according to Strohecker and Henning [22]. To total phenolic analysis content was made by the method proposed by Waterhouse [23]. The potential antioxidant activity was determined using DPPH (2,2-diphenyl-1-picryl hydrazyl), made in methanol/acetone solution, with some adaptations of Rufino et al. [24].

### 2.6. Microbiological Analysis

Microbiological analyses were performed to detect mold and yeast colony-forming units (CFU), according to the law [25]. The preparation of the samples and the dilutions was made as follows: 25 grams of each sample was weighed and transferred into a flask containing 225 mL of 0.1% sterile peptone water and homogenized for 2 minutes using the Stomacher; this initial dilution denominated (10^−1^). Serial dilutions 10^−2^ and 10^−3^ were then prepared. From each dilution 0.1 mL aliquots were removed and transferred to Petri dishes, in triplicate, in the of DRBC culture medium (Dichloran Rose-Bengal Chloramphenicol). The dishes were incubated for a period of 5 to 7 days at 25°C. The results were expressed as colony-forming units (CFU) per gram.

## 3. Results and Discussion

It was found that only the storage time has a significant effect on pH, titratable acidity (TA), soluble solids (SS), total sugar (TS), and total carotenoids (TC).  Table 2 contains the equations, R^2^, Fc, and Ft, statistically analyzed variables related to dietetic functional mixed cerrado fruit jam during storage.

Regression models at a 5% level of significance were established from the experimental results of the variables studied. The suitability of complete models can be verified (Table 2) by the coefficients of determination (R^2^), which explain between 70 and 93% of the total variance of the responses.

According to the results, it appears that for the parameters pH, SS, and TC (Table 2) there was no adjustment in the mathematical model, since their coefficients of determination were lower than 70%, meaning that the adjusted model did not fit the experimental data, showing averages of 4.15, 61%, and 470*μ*g /100g, respectively.

Damiani et al. [12] found that the pH of marolo and strawberry guava jam, during the first 6 months of storage, presented a slight decrease from 3.31 to 3.27 and after 12 months the pH increased to 3.33. The pH in the present study is classified as acidic (pH between 4.0 and 4.5) promoting an inhibitory effect on microorganism growth and increasing the useful life of alimentos.

Mesquita et al. [13] found a decrease in pH (3.9 - 3.7) in sugar-free guava jam during storage. Khouryieh et al. [26] report this pH value decrease behavior can be linked to dissociation of organic acids over time.

Policarpo et al. [6] analyzing umbu jam observed that during storage there was pH stability, a result similar to that of the present work. Rababah et al. [11] evaluated the cherry jam between 0 and 15 days and found pH and soluble solids values from 3.66 to 3.29 and 11.25% to 66.30%, respectively. Prati et al. [27] detected a pH of 3.45 in yacon, guava, and acerola mixed jam, without added sugars, a content lower than that found in this present study.

The variables titratable acidity, total sugars, and total phenolic were adjusted to the mathematical model, presenting determination coefficients between 70 and 93%.

**Figure 1:**
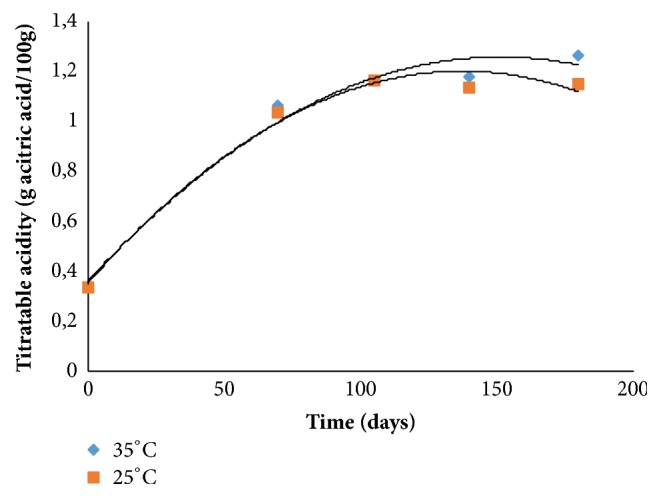
Titratable acidity behavior during storage.

It was observed that the titratable acidity increased from 0.41 to 1.2% during the storage period of 0-140 days (Figure 1), with a further reduction at 180 days. In preparing jams and jellies the acidity should be controlled and remain between 0.3 and 0.8%. When this acidity is above 0.8%, syneresis may occur, a fact that was detected in between 70 and 105 days of storage.

Mesquita et al. [13] evaluated sugar-free guava jam and found a sharp increase of acidity, of 1.2% to 1.9 during storage, values above those of this study.

**Figure 2:**
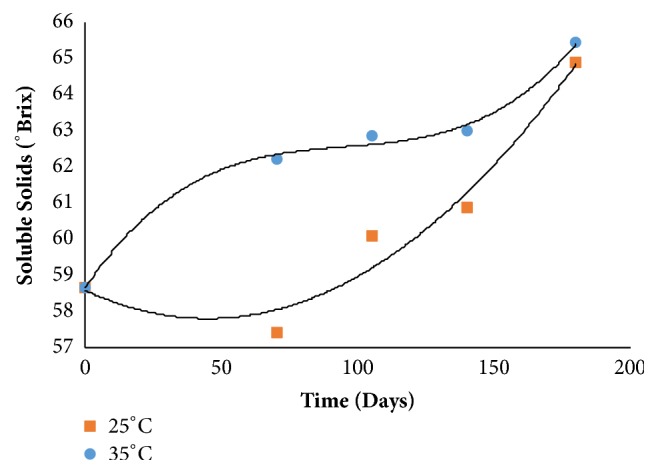
Total soluble solids during storage.

With respect to the variable soluble solids (Figure 2), there was a decrease up to 70 days (58 °Brix) with a subsequent increase of around 64 °Brix during storage in temperature of 25°C. At temperature of 35°C an increase of soluble solids until 65°Brix during 180 days of storage was observed (Figure 2).

Damiani et al. [12] observed that the soluble solids content ranged from 68.40 to 72.18 °Brix during storage for marolo and strawberry guava jam.

**Figure 3:**
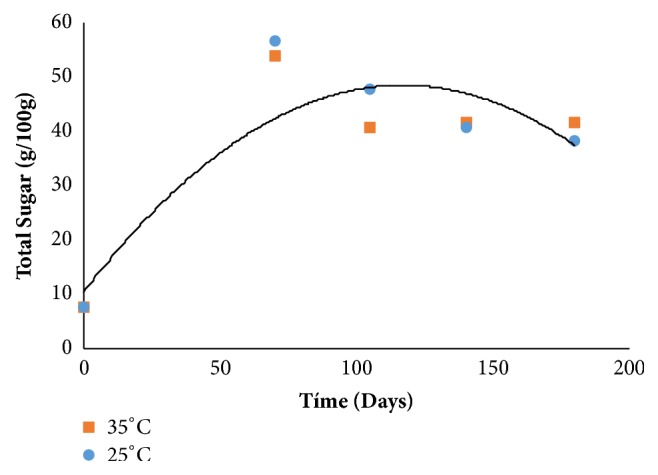
Total sugar behavior during storage.

With respect to total sugars (Figure 3) there was an increase up to 105 days (76% DM), with a decrease at the end of storage (57% DM), a behavior that can be associated with decreased humidity during storage [12].

Assis et al. [28] found average total sugar levels of 68.59% in cashew jam during storage (0-120 days). Zambiazi Chim and Bruscatto [16] evaluated 4 strawberry jam formulations (F1 conventional jam and F2, F3, and F4 “light” jellies) with different concentrations of sweeteners (saccharin, cyclamate, and cyclamate: saccharin) over a period of time (0, 60 and 120 days) and found that the conventional jam and “light” had average total sugar values of 62.21% and 44.7% during storage.

Phenolic compounds are potentially bioactive substances that naturally occur in plants and derived foods; scientific evidence has emphasized their beneficial role in health and disease prevention in humans [29].

For total phenolics, only the retention time was statistically significant. There was a marked decrease up to 140 days (320 mg/100g DM) with a slight increase at 180 days (380 mg/100g DM).

Phenolic compounds are directly related to the organoleptic characteristics of a particular product because this reduction may influence the changes in flavor, color, and aroma of the product.

According to Fennema [30], not only is the thermal energy the only factor that transforms bioactive agents in foods, but also treatment with use of acids associated with processing can promote the appearance of other phenolic compounds, such as the degradation of anthocyanins to phenolic acids. These decreases are due to the darkening of the product due to several oxidative reactions that occur during thermal treatment, such as oxidation of vitamin C, Maillard reaction, and carotenoid degradation, promoting dark pigments.

With regard to vitamin C and antioxidant activity, DPPH, there was no significant interaction between time and storage temperature. For vitamin C, there was no adjustment of the mathematical model for both temperatures (R^2^ =. 64%); however, for the DPPH, the mathematical model presented a coefficient of determination between 71 and 76% at temperatures 25 and 35° C, respectively (Table 2).

**Figure 4:**
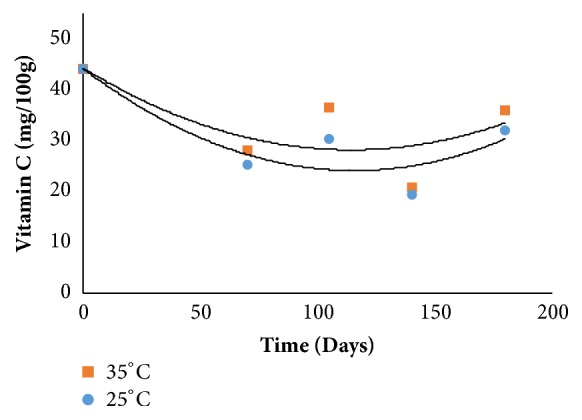
Vitamin C behavior during storage.

Regarding the vitamin results C, in Figure 4, a decrease can be observed during the 105 days of storage, a subsequent increase occurring until 180 days.

Importantly, although quantitatively the vitamin C loss was considered reasonable during storage, it was not enough to compromise the nutritional value of the product, since the mixed jam vitamin C content is comparable to foods that present average levels, ranging 30-50mg.100g^−1^ according to Ramful et al. [7].

Prati et al. [27] studied the vitamin C content in yacon, guava, and acerola jam during storage time (180 days) and found a loss of 42.7%. Patras et al. [10] evaluated the ascorbic acid content of strawberry jam stored at two temperatures, 4 and 15°C, and observed decrease in this parameter with the increase of temperature and storage time, leading to a ascorbic acid reduction percentage of 10% and 29.9% after 7 days and also found that after 28 days of storage this reduction percentage ranged from 49.7% to 70% at both temperatures, respectively.

**Figure 5:**
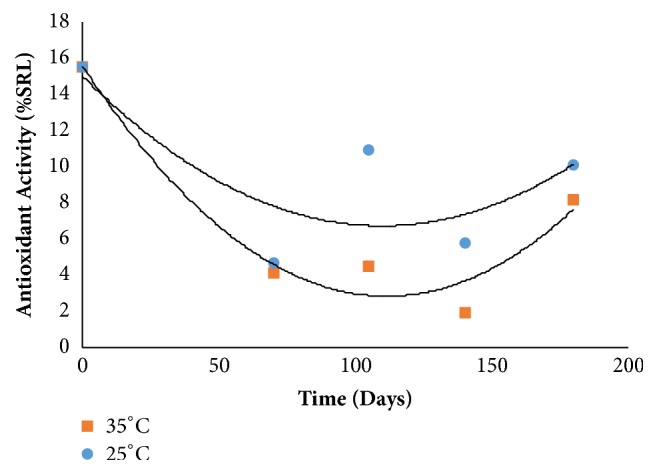
Antioxidant activity behavior during storage.

Regarding the antioxidant activity there was no effect of the interaction between storage time and temperature, where at both temperatures, 25 and 35°C, there was a decrease up to 105 days with subsequent increase at 180 days. At 25°C there was a less marked decrease than at 35°C (Figure 5)

Rababah et al. [11] evaluated cherry jam for a period of 15 days at different temperatures (25, 35, 45, and 55°C) and verified that there was a 50.72% decrease in antioxidant activity of the jam when compared to the initial value of this variable after processing and after the 15-day period, according to the cited temperatures, the average levels found were 42.07, 39.75, 20.83, and 10.68%, respectively.

Patras et al. [10] observed that there was degradation of bioactive compounds (ascorbic acid, anthocyanins, total phenolics, and antioxidant activity) in strawberry during storage for 28 days at 4 and 15°C; with the temperature increase, there occurred ascorbic acid degradation.

Regarding microbiological analyzes in mixed jam during storage, we observed that the findings presented in Table 3 are within the limits advocated by the legislation, which is 10^4^ CFU/g (colony-forming units), indicating that the jams obtained were in accordance with the hygienic standards [30].

Therefore, the mixed jam presented satisfactory health condition, meeting the sanitary standards established by RDC No. 12 of January 2, 2001, of the Secretary of Health Surveillance [25].

Similar results were obtained by Policarpo et al. [6] studying green umbu jam. Mesquita et al. [13] who evaluated the sugar-free guava jam also detected mold and yeasts values below the limit established by law.

## 4. Conclusions

The production of dietetic functional mixed cerrado fruit jam is an attractive option of using these fruits and is an alternative for processing of the cerrado fruits at harvest period. The storage time was the factor that most influenced the physicochemical changes in dietetic functional mixed cerrado fruit jam. The titratable acidity, soluble solids, and total sugar values increased and the values found for total carotenoids and total phenolics verified that there was a slight decrease during storage. As for vitamin C and antioxidant activity, their values showed a decrease at the two temperatures, 25 and 35°C, during storage. Regarding the microbiological analysis, we observed that there were fluctuations in yeast and filamentous fungi growth, but the results, during storage and at both temperatures, were within the standards required by law.

## Figures and Tables

**Table 1:** Formulation used in the production of dietetic functional mixed cerrado fruit jam.

**Ingredients**	**Concentration **[%]^**∗**^
Mixture of pulp	60.00
Fructooligosaccharide (P95 - oarfit®)	13.18
Polydextrose(commercial grade Nutramax®)	23.16
Citric Acid (commercial grade Nuclear®)	0.20
Potassium sorbate (commercial grade VETEC®)	0.20
Low-methoxylated Pectin – LMP(commercial grade Danisco®)	2.00
Locust bean gum*-LBG (commercial grade Danisco*®)	0.61
Carrageenan gum (commercial grade Danisco®)	0.61
Acesulfame-k (commercial grade Nutramax®)	0.01
Sucralose (commercial grade Nutramax®)	0.03
Total	100.00

^*∗*^In relation to the amount of the pulp.

**Table 2:** Regression equations adjusted for variables that do not present significant interaction between temperature and storage time (0, 70, 105, 140, and 180 days) and for variables that have a significant effect of the interaction between the mixed jam storage times (0, 70, 105, 140, and 180) and temperature (25°c and 35°C).

**Variable**	**Model estimator**	**R** ^**2**^	**Fc**	**Ft**
pH	Y = 4.11 + 0.0006*∗*x	0.49	1.84	3.97
TA	Y = 0.41 = 0.011*∗*x	0.89	2.75	3.97
SS	Y = 59.96 - 0.057*∗*x + 0.00047*∗*x^2^	0.55	1.77	3.97
TSS	Y = 16.87 + 1.14*∗*x - 0.005*∗*x^2^	0.70	0.44	3.97
TC	Y = 622.84 - 3.34*∗*x + 0.023*∗*x^2^	0.50	8.07	3.97
TP	Y = 2764 - 39.14*∗*x + 0.147*∗*x^2^	0.93	0.26	3.97
**Variable**	**Model estimator (T= 25**°**C)**	**R** ^**2**^	**Fc**	**Ft**

Vitamin C	Y = 56.42 - 0.35*∗*x + 0.001*∗*x^2^	0.64	5.26	3.97

DPPH	Y = 14.29 - 0.14*∗*x + 0.0006*∗*x^2^	0.71	21.68	3.97

**Variable**	**Model estimator (T= 35**°**C)**	**R** ^**2**^	**Fc**	**Ft**

Vitamin C	Y = 56.61 - 0.26*∗*x + 0.001*∗*x^2^	0.64	5.26	3.97

DPPH	Y = 14.86 - 0.21*∗*x + 0.0009*∗*x^2^	0.76	21.68	3.97

TA: titratable acidity; SS: soluble solids; TSS: total soluble sugars; TC: total carotenoids; TP: total phenolics; Fc: F calculated; Ft: F tabulated; antioxidant activity: DPPH.

**Table 3:** Mold and yeast count in the dietetic functional mixed cerrado fruit jam during storage.

Storage time (days)	Temperature (°C)	
	25	35
0	1.44x10^3^CFU/g	1.44x10 ^3^CFU/ g
70	1x10^3^ CFU/g	6x10^2^CFU/g
105	5.3x10^2^CFU/g	8.7x10^3^ CFU/g
140	7.6x10^3^CFU/g	5.1 x 10^2^CFU/g
180	3.3 x 10^3^ CFU/g	2.7 x 10^2^CFU/g

CFU: colony-forming units.

## Data Availability

All experimental data used to support the findings of this study are available from the corresponding author upon request.
